# Trial protocol for a multicenter randomized controlled trial to assess the efficacy and safety of intravenous ketamine for chronic daily headaches: the “KetHead” trial

**DOI:** 10.1186/s13063-023-07186-3

**Published:** 2023-03-01

**Authors:** Yasmine Hoydonckx, Mandeep Singh, Ian Gilron, James Khan, Samer Narouze, Albert Dahan, Kathryn Curtis, Xingshan Cao, Jamal Kara, Anuj Bhatia

**Affiliations:** 1 Department of Anesthesia and Pain Medicine, University of Toronto, Toronto Western Hospital, McL 2-405, 399 Bathurst Street, Toronto, Ontario M5T 2S8 Canada; 2 Departments of Anesthesiology & Perioperative Medicine and Biomedical & Molecular Sciences, Centre for Neuroscience Studies, School of Policy Studies, Queens University, Kingston, Canada; 3 Department of Anesthesia and Pain Medicine, University of Toronto, Sinai Health System, Toronto, Ontario Canada; 4 Department of Anesthesia and Pain Medicine, Western Reserve Hospital, Cuyahoga Falls, OH USA; 5 Department of Anesthesiology, Leiden University Medical Center, Leiden, The Netherlands; 6 Comprehensive Integrated Pain Program, University Health Network, Toronto, Canada; 7 Sunnybrook Health Sciences Centre, University of Toronto, Toronto, Canada

**Keywords:** Chronic daily headaches, Migraine, NMDA receptor, Ketamine, Quality of life, Pain

## Abstract

**Background:**

Chronic daily headaches (CDH) are common and associated with significant morbidity, poor quality of life, and substantial burden on the healthcare system. CDH tends to be refractory to conventional medical management and/or patients cannot afford expensive treatments. It is stipulated that CDH share a mechanism of central sensitization in the trigeminocervical complex, mediated by activation of the *N*-methyl-d-aspartate (NMDA) receptors. Ketamine, a non-competitive NMDA antagonist, has been used in the treatment of chronic pain, but its role in CDH has not been completely established. This trial aims to evaluate the effect of high-dose IV ketamine infusions (compared to placebo) on the number of headache days at 28 days post-infusion.

**Methods:**

A multicenter, placebo-controlled, randomized controlled trial will be conducted with two parallel groups and blinding of participants and outcome assessors. The study will include 56 adults with a CDH diagnosis as per ICHD-3 criteria. Participants will be randomized (1:1) to either ketamine (1 mg. kg^−1^ bolus followed by infusion of 1 mg. kg^−1^. h^−1^ for 6 h) or placebo (0.9% saline in the same volume and infusion rate as the trial medication) bolus and infusion for 6 h. The impact on the number of monthly headache days, headache intensity, physical activity, mood, sleep, quality of life, analgesic consumption, and adverse effects will be recorded at baseline, immediately post-infusion, and from 1 to 28 days, 29 to 56 days, and 57 to 84 days after the infusion

**Discussion:**

Despite advancements in treatment, many patients continue to suffer from CDH. This trial investigates whether high-dose IV ketamine infusions can effectively and safely improve the CDH burden as compared to a placebo infusion. This treatment could become a safe, affordable, and widely available option for patients living with refractory headache.

**Trial registration:**

ClinicalTrials.gov NCT05306899. Registered on April 1, 2022

**Supplementary Information:**

The online version contains supplementary material available at 10.1186/s13063-023-07186-3.

## Administrative information

World Health Organization Registration Data Set

**Table:** 

Title	The efficacy and safety of intravenous ketamine for chronic daily headaches: Trial protocol for a randomized controlled “KetHead” trial
Primary registry and trial identifying number	ClinicalTrials.gov NCT05306899
Other protocol identifier	21-5523
Date of registration in primary registry	April 1, 2022
Protocol version	Version 3.0 dated 16Aug2022
Funding	Canadian Pain Society and Pfizer Canada
Author details	**Yasmine Hoydonckx MD MSc FIPP** **Mandeep Singh MBBS MD MSc FRCPC** **Jamal Kara MSc CCRP** **Anuj Bhatia MBBS MD PhD FCCPC** Department of Anesthesia and Pain Medicine, University of Toronto, Toronto Western Hospital, Toronto, Ontario, Canada **Ian Gilron MD MSc** Department of Anesthesiology and Perioperative Medicine, Queens University, Kingston, Canada **James Khan MD MSc FRCPC** Department of Anesthesia and Pain Medicine, University of Toronto, Mount Sinai Hospital, Toronto, Ontario, Canada **Samer Narouze MD PhD** Department of Anesthesia and Pain Medicine, Western Reserve Hospital, Cuyahoga Falls, Ohio, USA **Albert Dahan MD PhD** Department of Anesthesiology, Leiden University, Leiden, The Netherlands **Kathryn Curtis PhD, C. Psych.** Comprehensive Integrated Pain Program, University Health Network, Toronto, Canada **Xingshan Cao PhD** Sunnybrook Health Sciences Centre, University of Toronto, Toronto, Canada
Primary sponsor	Department of Anesthesia and Pain Management University Health Network 399 Bathurst Street, 2-McL Toronto, ON M5T 2S8 Tel: (416) 603-5800 ext. 6237 Fax: (416) 603-6494
Contact for public queries	Department of Anesthesia and Pain Management Toronto Western Hospital, University Health Network, 399 Bathurst Street Toronto
Contact for scientific queries	Principal Investigator: Yasmine Hoydonckx MD, MSc, FIPP Toronto Western Hospital, University Health Network 399 Bathurst Street, 2-McL Toronto, ON M5T 2S8 yasmine.hoydonckx@uhn.ca Tel: (416) 603-5800 ext. 6237 Fax: (416) 603-6494 Contact for scientific queries: Jamal Kara MSc, CCRP Trial Coordinator Department of Anesthesia and Pain Management Toronto Western Hospital, University Health Network 399 Bathurst Street, 2-McL Toronto, ON M5T 2S8 Jamal.kara@uhn.ca kawalpreet.singh@uhnresearch.ca Tel: (416) 603-5800 ext. 6237 Fax: (416) 603-6494
Role of sponsor	The sponsor or funders have no role in the trial design, collection, management, analysis, and interpretation of the data; writing of the report; and the decision to submit the report for publication.

## Introduction

### Background and rationale

The prevalence of primary chronic daily headaches (CDH) is 5% in the population and is associated with significant morbidity, a poor quality of life, and a substantial burden on the healthcare system [1]. Important subtypes of CDH include chronic migraine, chronic tension-type headache, hemicrania continua, new daily persistent headache, and medication overuse headache [2–5]. Many patients with CDH do not respond to conventional medical management that includes medications, nerve blocks, and psychological approaches, and adverse events with these treatments are also common [6, 7]. Although onabotulinum-toxin A and calcitonin gene-related peptide (CGRP) antagonists are promising new treatments for CDH, these are expensive and their long-term effects are unclear. There is an urgent need for efficacious, safe, inexpensive, and widely available treatments for patients with CDH.

The subtypes of CDH share central sensitization, an increase in neuronal excitability secondary to repetitive stimulation of the nociceptive C-fibers (“wind-up”) in the trigeminocervical complex (TCC) and the brain, as a common mechanism [8–10]. The TCC contains major relay neurons for nociceptive afferent input from the meninges and cervical structures and its sensitization is a key feature of CDH. Central sensitization is mediated primarily by *N*-methyl-d-aspartate (NMDA) receptors. Activation of the NMDA receptors also plays a major role in ongoing pain, opioid-induced hyperalgesia, and mood dysregulation [10]. This sensitization can be reversed on a long-term basis by the blockade of NMDA receptors by non-competitive antagonists such as ketamine [8–10]. Intravenous (IV) ketamine in high doses (0.4 to 1 mg. kg^−1^ .h^−1^) has been used to treat NMDA receptor-driven neuropathic pain syndromes [8].

Recently, ketamine administered through IV and other routes has been investigated for its role in refractory headaches in observational studies [11–13] and a few small randomized controlled trials (RCT) [14–16]. This evidence suggests analgesic benefit, but it is of low quality, and there is considerable heterogeneity across the studies in terms of indications, dose, and route of administration of ketamine and the pain-associated domains that have been assessed [17]. Recent evidence also shows that infusions of ketamine 1 mg kg^−1^ h^−1^ are associated with greater and sustained pain relief as compared to only a bolus and or low-dose infusion regimens [8, 10, 17].

#### Rationale

CDH poses a significant burden on patients, healthcare systems, and society. IV ketamine infusion, an intervention that is widely available and scalable, can treat CDH by reversing the NMDA receptor-mediated sensitization of the TCC. This KetHead trial is the first multicenter, placebo-controlled, parallel-group randomized trial with blinding of participants and observers and with the goal of comprehensively assessing the effect of high-dose IV ketamine infusion (1 mg. kg^−1^. h^−1^ for 6 h) on the frequency and intensity of headaches, mood, activity, sleep, quality of life, and safety of ketamine for 84 days after the interventions, as compared to placebo.

### Objectives

The following is the primary objective:

1. To determine the number of days with headache in the first 28 days after the ketamine infusion

The following are the secondary objectives:

1. To determine the number of days with headache from 29 to 56 days and 57 to 84 days after the infusion
2. To determine the average intensity and duration of headaches headache from 1 to 28 days, 29 to 56 days, and 57 to 84 days after the infusion
3. To determine the number of days until recurrence of headache after infusion
4. To determine the daily requirement for analgesics at 28, 56, and 84 days after the infusion
5. To determine the quality and duration of sleep at 28, 56, and 84 days after the infusion
6. To determine the average daily physical activity and interference in functioning from CDH at 28, 56, and 84 days after the infusion
7. To assess mental health domains (depression, anxiety, and pain catastrophizing) at 28, 56, and 84 days after the infusion
8. To assess the quality of life and participants’ impression of change at 28, 56, and 84 days after the infusion
9. To assess the prevalence of adverse effects at 7, 28, 56, and 84 days after the infusion

### Hypothesis

The primary hypothesis of this trial is that patients with CDH undergoing high-dose ketamine infusion will report a decrease in the number of headache days in the first 28 days after the infusion as compared to the saline infusion (placebo).

The secondary hypotheses of this trial are that patients with CDH undergoing high-dose ketamine infusion will report a decrease in the number of headache days and daily requirement of analgesics and show an improvement in quality and duration of sleep, physical activity, mental health, and quality of life as compared to the saline infusion (placebo).

### Trial design

The KetHead trial is designed as a multi-center, placebo-controlled, superiority randomized controlled trial with two parallel groups and blinding of participants and outcome assessors. Randomization will take place upon participant enrollment. Randomization will be to one of two arms, with a 1:1 allocation and permuted blocks of six. The randomization sequence will be computer-generated, and allocation concealment will be ensured by sequentially numbered, sealed opaque envelopes. This process will be carried out by an independent research coordinator, who is not involved in the recruitment process or conduct of the trial. An unblinded pharmacist will dispense the trial medication to an unblinded and delegated healthcare provider who will then prepare the trial medication according to protocol and then present it to a blinded physician, who will administer the trial medication according to the protocol. A blinded member of the research team will perform an outcome assessment. Treating physicians, participants, close contacts, trial coordinators, and primary outcome assessors will be blinded to treatment allocation.

## Methods

### Trial setting

The trial will be conducted at two sites: Toronto Western Hospital (University Health Network) and Sinai Health System, both in Toronto, Ontario, Canada.

### Eligibility criteria

The inclusion and exclusion criteria are listed in Table 1. After enrollment, bloodwork to rule out hepatic and/or renal impairment is ordered if no recent (within 12 months) information is available. Additionally, on the day of the intervention, a pregnancy test is performed for each female participant less than 50 years of age.

**Table 1:** Inclusion and exclusion criteria

***Inclusion criteria*:** 1. Age 18 years or older 2. CDH diagnosis according to the International Classification of Headache Disorder – 3rd edition [18] 3. Normal liver and kidney function ***Exclusion criteria*:** 1. Pregnant or breastfeeding participants 2. Pre-existing renal impairment: stage 4 chronic kidney disease 3. Pre-existing liver impairment, defined as alanine transaminase (ALT) and/or aspartate aminotransferase (AST) greater than two times the upper normal limit, an elevated total bilirubin (TB), an elevated alkaline phosphatase (AP), an elevated international normalized ratio (INR) for prothrombin time, and/or an elevated blood urea nitrogen (BUN) 4. Chronic benzodiazepine or antipsychotic medication use 5. History of a cerebrovascular event 6. Significant and untreated hypertension and severe cardiac decompensation 7. Pheochromocytoma 8. Hyperthyroidism 9. Glaucoma 10. Concomitant use of strong CYP2B6 or CYP2C8 inhibitor 11. Allergy or intolerance to ketamine 12. Any significant cognitive or language barriers that impede participation 13. Use of calcitonin gene-related peptide antagonists in one month or onabotulinum-toxin A in 3 months prior to the trial medication infusion, that is associated with greater than 30% reduction in headache intensity or number of migraine days 14. Active diagnosis of post-traumatic stress disorder 15. Active diagnosis of substance use disorder 16. Daily oral morphine equivalent (OME) of 80 mg or higher	

### Interventions

#### Interventions common to both arms

**Fig. 1:**
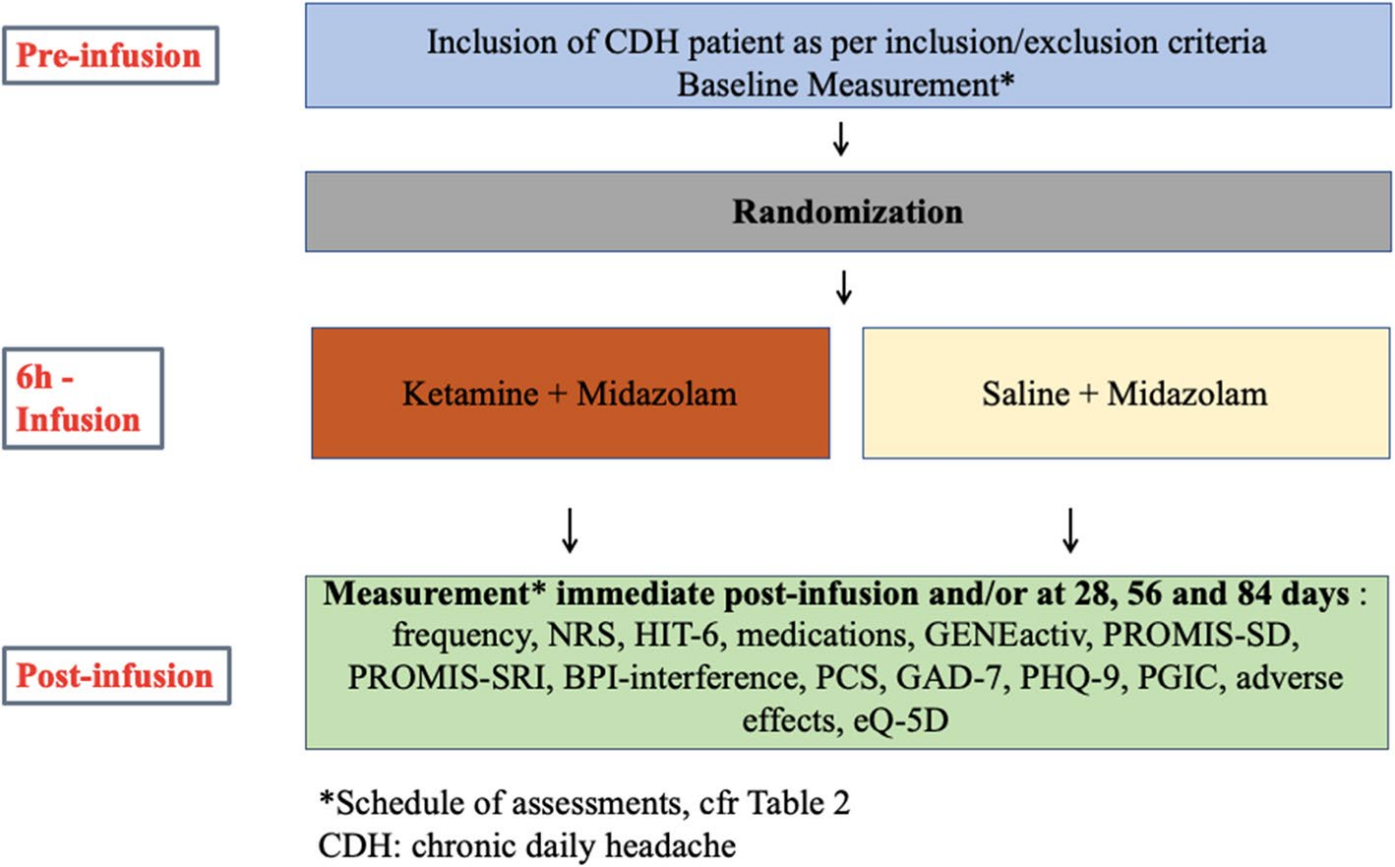
Flow diagram for the KetHead RCT

Trial participants will receive the infusion at the pain intravenous infusion unit at Toronto Western Hospital (Toronto, Ontario, Canada), under cardiorespiratory monitoring, supervised by an anesthesiologist. At the start of the infusion, all participants will receive IV midazolam 0.04 mg kg^−1^ (maximum 3 mg) and subsequently 0.01–0.02 mg kg^−1^ every hour to keep participants in a sedated but arousable state (Ramsay Sedation Scale score 3 or 4) [19] in an attempt to blind the participants and assessors to trial group allocation. Eight milligrams of ondansetron and 8 mg of dexamethasone will be administered to all participants to prevent nausea and vomiting, and 5000 units of heparin will be given subcutaneously to prevent thrombo-embolic events. All medications will be administered by a trained and board-certified anesthesiologist (Fig. 1).

##### Intervention group

For individuals randomized to the IV Ketamine group, 1 mg. kg^−1^ bolus will be given. To deliver this dose, a volume of 0.1 mL.kg^−1^ will be administered from a syringe of 10 mL with 10 mg/mL of ketamine. This will be followed by an infusion of 1 mg. kg^−1^ .h^−1^ (ketamine diluted in saline to 2 mg. mL^−1^; so an infusion rate of 0.5 mL .kg^−1^. h^−1^) for 6 h.

##### Control group

For individuals randomized to the saline infusion group, an IV bolus of 0.9% saline (0.1 ml. kg^−1^) will be given. This is followed by an infusion of 0.5 mL. kg^−1^. h^−1^ of saline for 6 h. The volume of bolus and rate of the infusion will be the same as that of a ketamine infusion for the body weight to prevent unblinding of participants and assessors.

We will use an approved Health Canada-marketed version of ketamine hydrochloride for this trial. The trial medication will be prepared by appropriately qualified health personnel in order to ensure the integrity of the trial protocol and to maintain blinding. The pharmacist dispensing the trial medication will not be blinded but they will not interact with the trial participants at any stage. When a patient is randomized, the delegated clinical nurse will check the corresponding treatment number (as assigned through an opaque sequentially numbered allocation envelope) and provide a prescription to the pharmacist to dispense the trial medication. In the event a subject is randomized but never administered any trial medication, the treatment will not be reassigned. Trial medication preparations are subject-specific only and will not be administered to another patient.

Trial personnel will assess the patient and collect data throughout their enrollment in the trial.

During the trial, participants will be instructed to use a pain and migraine diary for the collection of data on migraine days, pain scores, and rescue pain medication during the first 84 days after the infusion.

Participants will be assessed for the collection of outcomes immediately after the infusion and during the first 84 days after the infusion.

Participants in both arms will wear an actigraphy device (Additional file 1: Appendix 1) to assess the quality and duration of sleep and activity for 1 week prior to the trial medication infusion (for collection of baseline data) and starting on the day of infusion for 1 month after the infusion to longitudinally assess the impact of the trial treatments on sleep and activity.

#### Modifications

Discontinuation of the trial procedure will occur at the request of the participant. Any participant who experiences a serious adverse event at any point will immediately discontinue participation in the trial. The timing and reason for the discontinuation of the trial will be recorded.

#### Concomitant care

There will be no restrictions or changes to the baseline home medication, but participants will be advised to avoid the introduction of new analgesic agents in the post-infusion period.

#### Rescue medication and risk management

Any other pain medications provided during the infusion are considered rescue therapy. Participants will be given symptomatic treatment, and standard care will be provided. If any side effects or complications arise due to the use of trial medications, emergency unblinding will only occur when knowledge of the intervention is essential for medical care as determined by the principal investigator. In the event of emergency unblinding of one or more research team members, the timing, reason, and personnel involved will be recorded in the case record form (CRF), and blinding will be maintained for as many other trial personnel as possible.

### Outcomes

#### Primary clinical outcome

The between-group difference in the number of headache days in the first 28 days after the infusion is the primary outcome of this trial. A headache day is defined as a day in which the headache lasts 4 or more hours or a headache of any duration for which abortive treatment (anti-inflammatories, triptans, ergot derivatives, opioids) is taken. During the trial, participants will be asked to keep track of their headache days in a diary.

#### Secondary outcomes

1. Number of headache days in periods from 29 to 56 days and 57 to 84 days after the infusion; the between-group difference in the number of headache days from 29 to 56 days and 57 to 84 days after the infusion.

The following will be assessed at baseline and from 1 to 28 days, 29 to 56 days, and 57 to 84 days after the infusion:

1. Pain intensity: average intensity of headaches as measured on an 11-point Numerical Rating Scale (NRS; 0–10) [20]. During the trial, participants will be asked to rate their daily average pain level in a diary. The baseline headache intensity will be calculated as the mean of the daily pain score in the 28 days before the trial infusion.
2. Duration of headache episodes: the duration of the (daily) headache will be measured in hours in the pain diary. The baseline headache duration will be calculated as the mean of the duration of daily headaches in the 28 days before the trial infusion.
3. Time until the new onset of headache: determine the average number of days until the recurrence of headache after the trial infusion.
4. Sleep duration and quality: average daily duration of sleep as measured by the actigraphy wearable device (for 28 days after the infusion).
5. Sleep quality: quality of sleep as measured by the Pittsburgh Sleep Quality Index (PSQI) [21].
6. Physical activity: average daily physical activity as measured by the actigraphy wearable device (at 28 days after the infusion) and Brief Pain Inventory (BPI) [22].
7. Functioning: impact of headaches on functioning as measured by the Headache Impact Test (HIT-6) [23] score and Migraine Disability Assessment (MIDAS) [24].
8. Psychological health: emotional function as measured by Generalized Anxiety Disorder-7 items (GAD-7) [25], Pain Catastrophizing Scale (PCS) [26], and Patient Health Questionnaire for Depression-9 items (PHQ-9) [27] instruments.
9. Quality of life: health-related quality of life measured by EuroQol-5 dimensions, 5-item questionnaire (EQ-5D-5L) [28].
10. Participant satisfaction: participant satisfaction as measured by the Participant Global Impression of Change (PGIC) [29].
11. Adverse effects: adverse effects of ketamine as measured on the Clinician-Administered Dissociative States Scale (CADSS) [30] and Bowdle [31] questionnaires at end of the infusion.
12. Analgesic consumption: analgesic consumption will be measured by intake frequency and doses of headache-specific (abortive and prophylactic) medication and average daily oral morphine equivalent (OME) in milligrams.

### Participant timeline

**Fig. 2:**
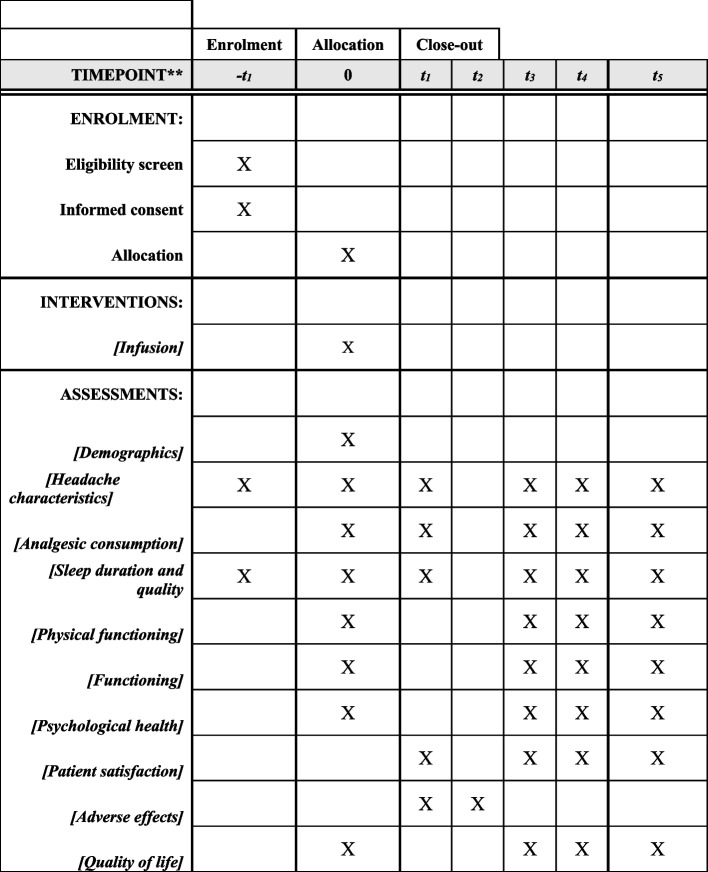
Schedule of enrollment, interventions, and assessments. -*t*1, 28 days before infusion; 0: day of infusion; *t*1, end of infusion; *t*2, at 7 days post-infusion; *t*3, at 28 days post-infusion; *t*4, at 56 days post-infusion; *t*5, at 84 days post-infusion

A 12-week trial during which the effect of high-dose ketamine infusion is compared to the saline infusion (placebo). The target of this trial is to demonstrate superiority. Outcomes will be assessed immediately after infusion and at 28 days, 56 days, and 84 days after infusion (Fig. 2).

The screening for eligibility to participate in this trial and the informed consent occurs within 3 to 6 months before the intervention (Additional file 2: Appendix 2). The trial starts a month prior to the day of the infusion, as the patient will start tracking the number of headache days and the daily pain score 28 days prior to the infusion date. This baseline headache diary will be collected on the day of the infusion. On that same day, the participant will fill out the validated questionnaires (completing baseline assessment). Baseline data on sleep and physical activity will also be collected using actigraphy over 1 week prior to the trial infusion. Scheduled follow-up visits at 28, 56, and 84 days after the trial infusion will be performed in-person or virtually (video/phone) to assess trial-relevant outcomes.

### Sample size

A difference of 3 headache days (standard deviation (SD) of 3.8) in the frequency of headache days between the two groups in the 28 days after the trial infusion is considered as a minimum clinically important difference (MCID) [32] for this trial. A sample size of 25 participants in each group would have over 80% power to detect this difference between the 2 groups using a two-sided alpha of 0.05. Assuming a 10% drop-out rate 1 month after the infusions, 28 participants will be enrolled in each arm. This sample size is also robust to capture a key secondary outcome for this trial—a MCID of 1.0 (SD 1.3) in the mean 0–10 NRS score for average intensity of headaches in the first 28 days after the infusions (power of over 80% with a two-sided alpha 0.05) [14].

### Recruitment

#### Feasibility of recruitment

Participants will be recruited from the Comprehensive Integrated Pain Program – Interventional Pain Service (CIPP-IPS) clinic at TWH or Wasser Pain Clinic at Sinai Health System (both in Toronto, Ontario, Canada). About 100 patients with CDH are treated in these centers each year, out of which 75 are likely to be eligible for participation in this trial. The trial will be discussed with all eligible participants. It is expected that 50% of all eligible participants (37 over 12 months) would consent to participate. Therefore, it is estimated that the enrollment of 56 participants for this trial will be completed in 18 months.

#### Recruitment strategy

The primary investigator will introduce this trial to pain physicians and neurologists who refer patients with CDH to the two trial sites. They will be encouraged to consider referring patients with CDH eligible for this trial.

## Assignment of interventions

### Randomization, sequence generation, and allocation concealment

Upon participant enrollment, patients will be randomized to one of two arms, with a 1:1 allocation and random permuted blocks of six. The randomization sequence will be computer generated by a member of the research team. The treatment allocation will be performed by an independent research coordinator not otherwise involved in the study, who will ensure allocation concealment by sequentially numbered, sealed opaque envelopes. This allocation sequence will be provided to the unblinded pharmacist

### Blinding

#### Blinding mechanism

A delegated unblinded healthcare provider with the appropriate qualifications will retrieve the trial medication of the unblinded pharmacist and prepare the trial medication. This healthcare provider will provide it to the blinded physician and blinded research team on the day of infusion. Participants, physicians, and all outcome assessors will all be blinded during the conduct of the trial and statistical analysis. Ketamine and saline are both clear liquids with no discernable smell. The use of IV midazolam during the infusion, which generates a sedated but arousable state (Ramsay Sedation Scale score of 3 or 4) [19] will increase the probability of blinding the participants and outcome assessors to group allocation.

#### Emergency unblinding

Emergency unblinding will only occur when knowledge of the intervention is essential for patient care as determined by the principal investigator or the Data Safety Monitoring Board. In the event of emergency unblinding, the timing, reason, and personnel involved will be recorded in the CRF, and blinding will be maintained in as many other trial personnel as possible.

## Data collection

### Data collection methods

All data will be collected on paper case record forms and then entered manually by trial personnel in duplicate, into a pilot-tested, password-protected, electronic database (Microsoft Excel platform).

Our data collectors are experienced research assistants who have been trained in Good Clinical Practice (GCP). There will be regular meetings between the data collectors and the trial principal investigator involved. We will use validated questionnaires for assessing pain and related domains. Patients will be followed closely at dedicated time points to promote retention and complete follow-up.

#### Baseline data

Data captured at baseline will include demographic information (patient age, gender, height, weight, comorbidities), headache-related information (type of headache, duration of pain, duration, and number of headache days and intensity in 28 days before infusion), vital signs (heart rate, blood pressure), medication and analgesic intake, quality of sleep, mood, and activity.

#### Infusion data

Data captured during and immediately after the infusion will include the intensity of headache (NRS), global improvement (PGIC), and adverse effects (Bowdle, CADSS)

#### Follow-up data

Data will be captured at 28, 56, and 84 days after the infusions and data will also be collected by the actigraphy device for the first 28 days after the infusion.

### Data management and confidentiality

All trial data will be checked for errors and missing data on a regular basis. Trial data from the actigraphy device and the app will be exported as a comma-separated values (CSV) file for analysis. It will be locally synced on an institutional research laptop. Participants’ contact information and identifying information will be encrypted and stored in a separate database. Identifying information will be deleted once follow-up is completed. The Institutional Research Ethics Board (IREB) audits trial records and collected personal health information to verify that the information collected for the trial is correct and to make sure the trial is following institutional regulations.

Completed questionnaires collected during the trial visits will be labeled with a unique trial code. No patient names will be used. The information that links the unique trial code to the participant will be kept in a secure area in our institute, and it will be distinct from the participants’ trial data. This information can only be accessed by research team members who are involved in the trial.

All records and documents pertaining to the trial will be retained by the trial sites for 25 years from the completion of the trial, as per Health Canada regulations.

In the event of personal health information disclosure to an unauthorized party, the following will be done: any further release of information will be stopped, as much information as possible will be retrieved, IREB privacy Offices will be contacted, and further actions would be taken based on their recommendations.

### Statistical methods

The normality of the trial data will be assessed, means and standard deviation (or median and interquartile ranges, as appropriate) will be used to summarize continuous data, and frequencies and proportions will be used for categorical data. To compare data between the 2 groups, categorical outcomes will be analyzed with the chi-square test and continuous variables will be analyzed with independent *t*-tests (or Wilcoxon rank sum test). A baseline-adjusted ANCOVA model will be employed for all outcome variables of interest for which a baseline is recorded in the 28 days interval prior to treatment. In addition, for the primary outcome, an additional ANCOVA model will be employed adjusting for baseline headache days and the following variables that are strongly associated with this outcome: number of headache years, depression/anxiety, intensity of headache, and opioid intake. Estimates of differences in means (with 95% CI) at 28, 56, and 84 days will be provided for continuous outcomes. A Cox proportional hazards model will be built to investigate the effect on variables on the time to a specified event. Missing primary outcome data will not be imputed. A sensitivity analysis assuming the participants lost to follow-up showed no improvement (i.e., they had no change in headache frequency) will then be performed. Group comparisons will be performed in responders (defined as a reduction in pain by 3 or more headache days in 28 days from baseline at 28 days after the infusions) versus non-responders, participants on Botox/CGRP antagonist treatment versus none, comparison across different subtype of CDH, and baseline frequency and average intensity of headaches. An intention-to-treat analysis approach will be used. SAS Studio Edition will be used to conduct the analyses.

## Data monitoring

### Formal committee

An independent Data Safety Monitoring Board (DSMB) whose members have experience in clinical trials has been formed. Monitoring and reporting of adverse events will be conducted throughout the whole trial. If there are concerns about the quality of care, and or the safety of any interventions, the committee will have the authority to stop the trial subject to evaluation by the UHN Research Ethics board.

### Interim analysis

There will be no planned interim analysis.

### Safety/harms

Monitoring and reporting of adverse events will be conducted from the time of consent to trial entry until the end of the trial. Adverse events will be documented in the patient file and electronic case report form and reported to the UHN REB.

#### Auditing

Formal audits will be performed at the request of the DSMB or UHN REB. Representatives of the IREB and Health Canada may look at the trial records and at personal health information to verify that the information collected for the trial is correct and to make sure the trial is following proper laws and guidelines.

The sponsor must ensure that investigator(s)/institution(s) will permit trial-related monitoring, audits, Institutional Review Board/Independent Ethics Committee (IEC) review, and regulatory inspection(s), providing direct access to source data/documents.

## Ethics and dissemination

### Research ethics approval

This trial will be conducted in accordance with the ethical principles outlined in the Declaration of Helsinki, the protocol, Good Clinical Practice guidelines, and applicable regulatory requirements. The trial is approved by REB of UHN and Sinai Health System, and Health Canada. Full written informed consent will be obtained from the participants by the research team (Additional file 2: Appendix 2). This trial has been registered on the ClinicalTrials.gov website (NCT05306899), and the results will be reported per SPIRIT guidelines. Before any changes to the trial are implemented, besides those to eliminate an immediate hazard to trial participants, an amendment to the trial will be submitted for review and approval by the IREB. There is no plan for ancillary studies.

### Criteria for subject withdrawal

Subjects have the right to withdraw from the trial at any time, without providing a reason. In the event that a subject decides to prematurely discontinue from the trial, they will be asked if they can still be contacted for further information. This will be documented accordingly. If the subject has already received the trial medication prior to withdrawal, the subject will be requested to be contacted for a safety follow-up visit.

When applicable, subjects should be informed of the circumstances under which their participation may be terminated by the investigator without their consent. The investigator may withdraw the subjects from the trial in the event of intercurrent illness, adverse events, lack of compliance, or at the request of the Institutional Review Board.

### Ancillary and post-trial care

No additional provisions will be made for post-trial care, and routine clinical care will be provided by the participant’s primary physician. If the patient suffers harm as a result of a study intervention, appropriate follow-up care will be provided as indicated.

### Dissemination policy

Trial results will first be disseminated at the local, national, and international conferences and submitted for publication in a peer-reviewed journal. Authorship will be in accordance with the International Committee of Medical Journal Editors guidelines.

## Discussion

Despite recent evolutions in the pharmacological and interventional management of CDH patients, many patients remain refractory to these treatments and/or cannot afford them. Recent evidence shows that infusions of ketamine in high doses and a prolonged duration of treatment could have a positive effect on refractory chronic pain [10], but the quality of evidence for efficacy in CDH is low [17].

This trial aims to investigate the efficacy of high-dose ketamine infusions for chronic daily headaches, by comparing a 6-h high-dose ketamine infusion to placebo. Fifty-six participants will be included and will be divided between the two groups. Participants and assessors will be blinded to the treatment allocation. Data on the frequency and intensity of headaches, mood, activity, sleep, quality of life, and safety of ketamine will be collected from baseline up to 84 days after the intervention.

This trial has several novelties. It is the first trial investigating the impact of a 6-h high-dose intravenous ketamine infusion on the intensity and frequency of CDH in a randomized, double-blinded, placebo-controlled manner. Furthermore, ketamine has been demonstrated to have an effect on sleep and mood and therefore improve the patient’s quality of life [33]. This will be the first trial assessing the impact of ketamine on multiple pain-associated domains by use of validated questionnaires and wearable technology.

Although recent data shows that infusions of ketamine of 1 mg.kg^−1^ .h^−1^ are associated with greater and sustained pain relief as compared to one-off bolus and low-dose infusion regimens, the adverse effects of ketamine are common and could limit dose escalation [8]. Ketamine is associated with adverse psychomimetic, cardiovascular, and gastrointestinal effects resulting from its activity on a variety of substrate receptors including NMDA, acetylcholine, opioid, monoamine, and histamine receptors [8]. The incidence of reported ketamine-induced adverse effects in the recent literature is high, regardless of the type of administration or dose, and has mostly included psychomimetic effects. Most adverse effects were mild and resolved after decreasing the dose or ending the treatment [17].

To limit the incidence of adverse effects in this trial and beyond, these infusions should only be performed by properly trained personnel in a highly monitored environment, which facilitates early detection of issues and prompt treatment. Secondly, all our participants will be treated with benzodiazepines and high-dose anti-emetics to limit the incidence of psychomimetic and gastrointestinal side effects.

This trial will help to identify if ketamine is an effective and safe alternative for headache treatment. The investigators hope the knowledge from this trial will be widely implemented to improve patients’ health and quality of life.

## Trial status

Protocol version and date: version 3.0, August 16, 2022

Date of start of recruitment: August 14, 2022

Estimated end of recruitment: October 1, 2023

Estimated trial completion date: February 1, 2024

## Supplementary Information

**Additional file 1: Appendix 1.** GENEActiv Actigraphy Monitoring.

**Additional file 2: Appendix 2.** Consent Form for Participation in a Research Study.

## Data Availability

Following the completion of data entry, only the trial investigators and trial coordinators will have access to the final trial dataset.

## Abbreviations

ADR(s): Adverse drug reaction(s)
AE(s): Adverse event(s)
BPI: Brief Pain Inventory
CADDS: Clinician-Administered Dissociative States Scale
CDH: Chronic daily headaches
CGRP: Calcitonin gene-related peptide
CM: Chronic migraine
DHD: Daily headache diary
EQ-5D: EuroQoL – 5 Dimension
EuroQol: Group measurement of health status
GAD-7: Generalized Anxiety Disorder-7 items
HC: Hemicrania continua
HIT-6: Headache Impact Test-6 item
IMP: Investigational medical product
MIDAS: Migraine Disability Assessment
MOH: Medication overuse headaches
MRP: Most responsible physician
NA: Not applicable
NDPH: New daily persistent headache
NMDA: *N*-methyl-d-aspartate
NRS: Numerical Rating Scale
OME: Oral morphine equivalents
PCS: Pain Catastrophizing Scale
PGIC: Patient Global Impression of Change
PHQ-9: Patient Health Questionaire-9
PI: Principal investigator
PSQI: Pittsburgh Sleep Quality Index
SAE: Serious adverse event
TCC: Trigeminocervical complex
TTH: Tension-type headache
TWH: Toronto Western Hospital
UHN: University Health Network
