# Supplementary material for: Trial protocol for a multicenter randomized controlled trial to assess the efficacy and safety of intravenous ketamine for chronic daily headaches: the “KetHead” trial
Source: Trials. 2023 Mar 1;24:155. doi: 10.1186/s13063-023-07186-3 (PMC9976458; doi:10.1186/s13063-023-07186-3)

**Appendix 1**: GENEActiv Actigraphy Monitoring

This is a non-invasive method of measuring activity and sleep patterns. A wrist-worn sensor that incorporates an accelerometer (GENEActiv®, Activeinsights, Cambridge, UK) measures activity, light and temperature continuously for up to one month. The raw data available for analysis is subject to algorithms available in an open data format. Use of actigraphy during trials of interventions for neuropathic pain, including SCS, allows decision-making based on objective data regarding improvement (or lack thereof) in physical activity and sleep during the trial.


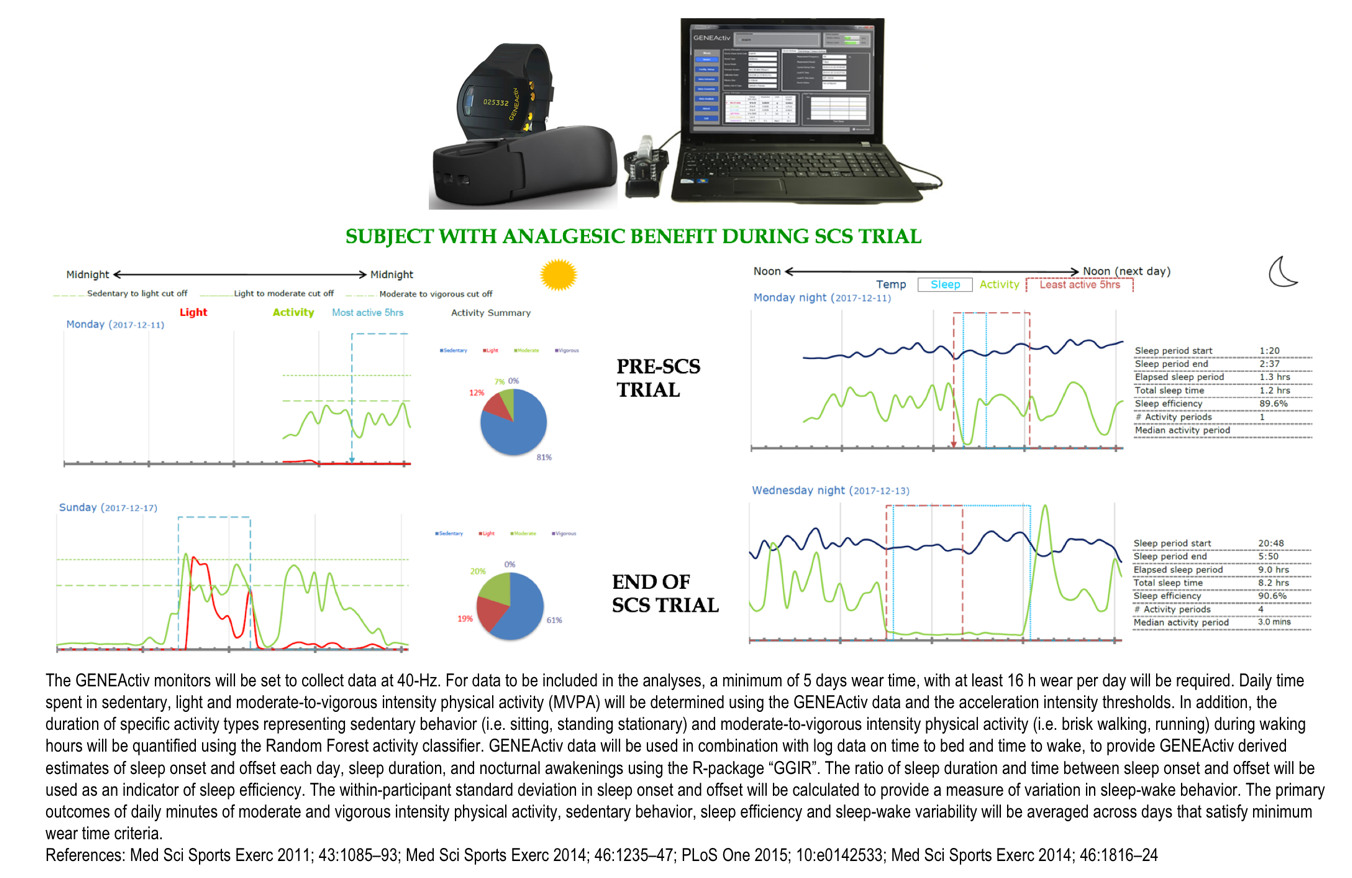

Supplement: Supplementary file 1 — Additional file 1: Appendix 1. GENEActiv Actigraphy Monitoring. [file 13063_2023_7186_MOESM1_ESM.docx]
