# Supplementary material for: Trial protocol for a multicenter randomized controlled trial to assess the efficacy and safety of intravenous ketamine for chronic daily headaches: the “KetHead” trial
Source: Trials. 2023 Mar 1;24:155. doi: 10.1186/s13063-023-07186-3 (PMC9976458; doi:10.1186/s13063-023-07186-3)
Supplement: Supplementary file 2 — Additional file 2: Appendix 2. Consent Form for Participation in a Research Study. [file 13063_2023_7186_MOESM2_ESM.pdf]

Consent Form for Participation in a Research Study

Title: A multi-center randomized controlled trial of efficacy and safety of intravenous ketamine for chronic daily headaches: The KetHead Study

Principal Investigator: Dr. Yasmine Hoydonckx
Staff Anesthesiologist
Department of Anesthesia and Pain Management

Study Coordinator: Kawalpreet Singh
Department of Anesthesia and Pain Management
416-603-5800 ext. 3959

Introduction

You are being asked to take part in a research study. Please read this explanation about the study and its risks and benefits before you decide if you would like to take part. You should take as much time as you need to make your decision. You should ask the study doctor or his/her designee to explain anything that you do not understand and make sure that all of your questions have been answered before signing this consent form. Before you make your decision, you may talk about this study with anyone you wish. Participation in this study is voluntary.

Background and Purpose

Over 5% of Canadians are affected by primary chronic daily headaches (CDH) such as migraines, tension-type headaches and medication overuse headaches. Many patients with CDH do not respond to conventional treatments such as nerve blocks and medications, where side effects can be quite common. Recent new medications advances for CDH can also be quite expensive with unclear long-term effects.

Recently, small observational studies focusing on the use of intravenous of ketamine administered to treat headaches have demonstrated effective pain relief. However, there is a need for studies that involve higher quality research to study the pain-relieving effect and optimal duration of high dose ketamine infusions in addition to its effect on related domains such as sleep and functional activity. Therefore, we propose to conduct a study to assess the efficacy and safety for high dose intravenous ketamine for chronic daily headaches (the KetHead Study).

Ketamine is an anesthetic drug. It is also known that when given in low doses, ketamine can improve pain control after surgery and chronic neuropathic conditions. It can be particularly helpful for patients whose pain is not adequately controlled with commonly used pain medications (refractory pain).

Health Canada has approved intravenous ketamine for medical use in the hospital, however there are currently no Health Canada approved indications for its use in managing pain from CDH. Therefore, the use of ketamine in this study is experimental. Experimental means Health Canada has not yet approved the use of ketamine for this indication, but they have approved its use in this research study. This study will look at how effective and safe ketamine is in controlling pain specifically for patients with chronic daily headaches.

Approximately 56 people will take part in this study at Toronto Western Hospital and Sinai Health System. We expect the study to last for about 18 months, however your participation in the study will be for 3 months

You are being asked to take part in this research study because you have currently been experiencing pain from CDH but have not experienced adequate pain relief from conventional medications or treatments.

If you decide to participate, the study doctor or his/her designee will be in contact with your regular health care provider throughout the time that you are in the study, if needed.

If you agree to be in the study, you will be booked for an infusion to assess the safety and efficacy of ketamine your headache-related pain by comparing the effects of each study drug (ketamine or placebo).

Study Design

This is a randomized double blinded study. If you decide to participate you will be "randomized" into one of the two study groups described below. Randomization means that you are put into a group by chance. It is like flipping a coin. Neither you nor your doctor will know or can choose what group you will be in. The pharmacist preparing the study medications will know which drug you will be receiving as determined by receiving a randomized envelope with what study drug group you will be assigned to. You will have a 1 in 2 chance of being placed in any group. In an emergency the doctor can get this information.

- If you are in the study drug group, you will receive ketamine at a dose of 1 mg / kg / hour for six hours.
- If you are in the control group, you will receive a placebo of 0.9% saline followed by a saline infusion at the same rate as the study drug group for six hours. Both saline and ketamine are colorless and will look identical so you will not be able to guess which study group you will be in.

A placebo is given in this study to reduce the chances of believing that your pain is getting better because you are receiving ketamine. However, both groups will receive IV midazolam at 0.04 mg / kg to start (at a maximum of 3 mg), and then 0.01-0.02 mg / kg / hour to allow you to be in a sedated, but arousable state so you will not know if you have received ketamine or saline solution. Pain control and regular postprocedural care will still be provided to you as per our standard practice.
Midazolam is a benzodiazepine that has sedative properties and is used to help reduce anxiety in patients. Midazolam can take up to 6 hours for the effects to wear off.

Additional drugs that may be administered during the infusion include ondansetron, dexamethasone, and heparin. Ondansetron and dexamethasone are meant to reduce any nausea that may occur during the ketamine infusion. Heparin is given to reduce the risk of Deep Vein Thrombosis (DVT) since patients will remain bedbound for the duration of the infusion.

In both groups, you will be asked to fill out a series of questionnaires during each visit and receive an accelerometer watch known as the GENEActiv. This watch will be given to you on the day of your infusion to assess your sleep and activity for approximately one month.

Study Procedures

If you agree to be in this study, you will be asked to sign this consent form. You will see the study doctor or his/her designee at a screening visit and again on the day of the infusion. There will be three study visits in total. At each of these visits the following assessments will be carried out:

Screening Visit (up to 28 days before scheduled day of infusion):

- The screening visit may take up to 1 hour.
- The following data will be collected from you:
  - Demographic information including age, sex, gender, height and weight
  - Current health conditions, medications, and information about your headaches
- You will also be asked to complete Pain Intensity score. This is a numeric rating scale to rate your pain from 0-10 over the past 24 hours
- You will also be asked to complete the following baseline questionnaires:
  - Pittsburgh Sleep Quality Index (PSQI) to assess the quality, duration, and interference with your sleep.
  - Patient Health Questionaire-9 (PHQ-9) to screen for the presence and severity of depression.
  - Generalized Anxiety Disorder 7-item (GAD-7) to screen for the presence and severity of anxiety
  - The EuroQol-5 dimension (ED-5D-5L) to assess your overall quality of life
  - The Pain Catastrophizing Scale (PCS) to assess your perception of pain and how it impacts your life
- Pregnancy Test - If you are a woman and can become pregnant, you will have a pregnancy test (via urine sample) before starting the study drug to be sure that you are not pregnant. If you are pregnant, you cannot participate in this study.

Day of infusion:

- You will be randomized to a study drug group (receiving Ketamine) or control group (receiving Placebo).
- You will receive the study drug or placebo infusion for 6 hours, and will be monitored by anesthesia assistants, nurses, and the study team until you are discharged.
- In both groups, you will also receive Midazolam, Ondansetron, and Dexamethasone to reduce the likelihood of anxiety and nausea during the procedure.
- The characteristics of your headaches will be monitored, along with your physical functioning, sleep, and medication intake.
- After the infusion, we will assess the adverse effects you may have experienced during the infusions using the Bowdle questionnaire and the Clinician-Administered Dissociative States Scale (CADSS).
- You will then be sent to the Day Surgery Recovery Unit to be discharged from the hospital. Upon discharge, you will also receive the GENEActiv accelerometer watch which will record your sleep patterns and movements in real time. This will be given on the day of the infusion and will be removed after one month of recording. The watch will not be used for the remainder of your participation in the study.
- You will need to arrange for transportation afterwards as you will not be able to drive due to the drugs administered during the infusion.

Follow ups (one week, 1-month, 2-months, and 3-months post-infusion):

Our study nurse will contact you one week after the infusion to assess for any side-effects from the infusion.

During the first three months after the infusion, we will ask you to keep a headache diary in which

you will note daily the presence/absence of a headache, its duration and severity and the rescue medication you might take.
You will be seen in the Comprehensive Integrated Pain Program – Interventional Pain Service (CIPP-IPS) clinic to assess your effect of the medication on your pain, health, and overall quality of life at 1-month, 2-months and 3-months post-infusion. Each visit will take about 30 minutes.

During this appointment, your medications and headache characteristics will also be assessed and you will repeat the same questionnaires as in your first visit in addition to the following:

- The Patient Global Impression of Change (PGIC) questionnaire to determine your overall satisfaction with the study drug

You will also be asked to remove the GENEActiv accelerometer watch during your 1-month post-infusion appointment.

It will take about 16 weeks to complete the study from signing the consent form to the follow up assessment.

Please see the table below for a summary of what will be done at each study visit.

Visit Schedules:

**TIMEPOINT (state unit)**	Baseline	Daily	Day of Infusion	1-week post-infusion	1-month post-infusion	2-months post-infusion	3-months post-infusion
**VISIT NUMBER:**	1		2		3		4
**ENROLLMENT:**	✓						
Eligibility screen	✓						
Informed consent	✓						
Allocation			✓				
**INTERVENTIONS:**							
Ketamine vs Placebo			✓				
**ASSESSMENTS:**							

Demographics	✓						
Headache characteristics	✓		✓		✓	✓	✓
Opioid Use	✓	✓	✓		✓	✓	✓
Pain Intensity	✓	✓	✓		✓	✓	✓
Emotional Functioning	✓						
Sleep	✓	✓			✓	✓	✓
Physical Activity	✓		✓		✓	✓	
Quality of Life	✓				✓	✓	✓
Side-Effects			✓	✓			

Risks Related to Being in the Study

Taking part in this study has risks. Some of these risks we know about. There is also a possibility of risks that we do not know about and have not been seen in humans to date. Please contact the study doctor or his/her designee if you have any side effects even if you do not think it has anything to do with this study.

Risks of Ketamine:

Ketamine is considered relatively safe in medical settings and has been used for decades for medical treatment. The following are the known side effects of ketamine when used as pain medication for surgical procedures.

- Cardiac effects (1% to 10%): Increased heart rate and blood pressure
- Central Nervous System effects (1% to 10%):
  - Dizziness and blurred or double vision
  - "Out-of-body" experience, floating sensation, hallucinations, dysphoria, vivid dreams
- Gastro-intestinal effects (1% to 10%):
  - Nausea and vomiting
  - Increased salivation and lacrimation
  - Abnormal liver function test: uncommon in case of short term use of ketamine.
- Other: itching (0.1% to 1%)

Risks of Ondansetron:

Ondansetron is considered safe in medical setting and has been used for decades for medical treatment to prevent and treat nausea. Side-effects are unusual and even less likely after a single dose treatment. The following are the known side effects:

- Nervous system (0.1-10%): drowsiness, sedation, transient involuntary muscle movements
- Gastrointestinal (1-10%) diarrhea, constipation, localized anal/rectal burning sensation, asymptomatic liver tests increase (AST/ALT)

- Ocular (1-10%): Transient blurred vision

- Cardiovascular (1% to 10%): Sensation of warmth/flushing, arrythmia, transient ECG changes including QT interval prolongation.
- Respiratory (1% to 10%): difficulty breathing, hiccups.
- Psychiatric (1% to 10%): Agitation and sleep disturbance
- Genitourinary (1% to 10%): Difficulties voiding
- Dermatologic (1% to 10%): Rash

- Other (1% to 10%): Injection site reaction, hypersensitivity reactions

Risks of Midazolam:
Midazolam has been used for decades to induce sedation in patients and is considered relatively safe in medical setting when administered under hemodynamic monitoring. The following are well known side-effect of midazolam when used as sedation medication for procedures.

- Nervous system (1% to 10%): Headache, oversedation, drowsiness, seizure (0.1-1%): difficulties with balance, temporary memory loss, agitation
- Cardiac (1-10%): low blood pressure
- Ocular (1-10%): involuntary movement of eyes
- Gastro-intestinal (1-10%): nausea, vomiting
- Respiratory (1-10%): difficulties breathing
- Other (1-10%): injection site tenderness

Risks of Dexamethasone:
Dexamethasone is considered safe in medical settings and has been used for medical treatment for decades. The most commonly occurring side effects include alteration in glucose tolerance, behavioral and mood changes, increased appetite, and weight gain; the incidence generally correlates with dosage, timing of administration, and duration of treatment. Given the fact that this will be a single treatment, the incidence of side-effects is deemed very low.

Side-effects that are more associated with prolonged use of dexamethasone

- Cardiovascular: slow or rapid heart rate, high blood pressure, edema
- Gastrointestinal: abdominal discomfort, nausea, bleeding, peptic ulcer, perforation of intestines
- Other: hypersensitivity reaction
- Endocrine: metabolic disorder (Cushingoid state)
- Metabolic: glucose tolerance and high blood sugar
- Ocular: glaucoma, blurred vision
- Musculoskeletal: dose-dependent muscle weakness and steroid muscle weakness, osteoporosis
- Dermatologic: rash

Risks of Enoxaparin:
Enoxaparin is considered safe for thrombo-embolic prevention in medical setting. Adverse reactions are rare and the mentioned below. In the case of your infusion, these risks are deemed even lower as this will be a single dose administration.

- Central nervous (0.1-1%): intracranial bleeding
- Cardiovascular (1-10%): bleeding, low platelets and red blood cells
- Respiratory (1-10%): difficulties breathing
- Gastro-intestinal (1-10%) asymptomatic elevation of liver tests, diarrhea, nausea
- Other (1-10%): injection site hematoma/pain/skin reaction
- Immunologic (1-10%): allergic reaction (less than 0.01%): immune-allergic thrombocytopenia

Benefits

You may not receive direct benefit from being in this study. Information learned from this study may help future headache pain treatment.

Reminders

It is important to remember the following things during this study:

- Ask your study team about anything that worries you.
- Tell study staff anything about your health that has changed.
- Tell your study team if you change your mind about being in this study.

Voluntary Participation:

Your participation in this study is voluntary. You may decide not to be in this study, or to be in the study now, and then change your mind later. You may leave the study at any time without affecting your care. You may refuse to answer any question you do not want to answer, or not answer an interview question by saying "pass".

We will keep you updated about new information that might be revealed during the course of the study, that might affect your decision to stay in the study.

Alternatives to Being in the Study:

You do not have to join this study to receive treatment for your condition. Alternative pharmacological options including botulinumtoxin injections and Calcitonin Gene Receptor Peptide (CGRP) antagonist medications could be provided to you by your pain physician. CGRP antagonist medications are a new class of drugs that can reduce chronic headache pain.

Withdrawal from Study:

The investigator may decide to remove you from this study without your consent at any time for any of the following reasons:

- The investigator decides that continuing in this study would be harmful to you.
- You are unable or unwilling to follow the study procedures.

If you decide to leave the study, you have the right to request withdrawal of information collected about you. Let your study doctor know.

If you are removed from this study, the investigator will discuss the reasons with you and plans will be made for your continued care outside of the study and it will have no effect on your care.

You can also choose to end your participation at any time without having to provide a reason. If you choose to withdraw, your choice will not have any effect on your current or future medical treatment or health care.

A description of this clinical trial will be available on *http://www.ClinicalTrials.gov.*

This website will not include information that can identify you. At most, the website will include a summary of the results. You can search this website at any time.

Costs and Reimbursement

There is no extra cost involved in this study. You will receive $30 to cover travel and parking expenses for participating in this study.

Rights as a Participant:

If you are harmed as a direct result of taking part in this study, all necessary medical treatment will be made available to you at no cost.

By signing this form, you do not give up any of your legal rights against the investigators, sponsor-investigator or involved institutions for compensation, nor does this form relieve the investigators, sponsor or involved institutions of their legal and professional responsibilities.

Confidentiality:

Personal Health Information

If you agree to join this study, the study doctor and his/her study team will look at your personal health information and collect only the information they need for the study. Personal health information is any information that could be used to identify you and includes your;

- Name,
- Age,
- Phone number
- New or existing medical records that includes types, dates and results of medical tests or procedures.

Your participation in this study will also be recorded in your medical record at this hospital. This is for clinical safety purposes.

Research Information in Shared Clinical Records

If you participate in this study, information about you from this research project may be stored in your hospital file and in the UHN computer system. The UHN shares the patient information stored on its computers with other hospitals and health care providers in Ontario so they can access the information if it is needed for your clinical care. The study team can tell you what information about you will be stored electronically and may be shared outside of the UHN. If you have any concerns about this, or have any questions, please contact the UHN Privacy Office at 416-340-4800, x6937 (or by email at privacy@uhn.ca)

The following people may come to the hospital to look at the study records and at your personal health information to check that the information collected for the study is correct and to make sure the study is following proper laws and guidelines:

- Representatives of the University Health Network (UHN) including the UHN Research Ethics Board.
- Representatives of Health Canada.

The study doctor will keep any personal health information about you in a secure and confidential location for 25 years as required. A list linking your study number with your name will be kept by the study doctor in a secure place, separate from your study file.

Study Information that Does Not Identify You

All information collected during this study, including your personal health information, will be

kept confidential and will not be shared with anyone outside the study unless required by law. You will not be named in any reports, publications, or presentations that may come from this study.

While the information and data resulting from this study may be presented at scientific meetings or published in a scientific journal, your identity will not be revealed.

Conflict of Interest

All of the people in this study have an interest in completing this study. Their interests should not influence your decision to participate in this study. You should not feel pressured to join this study.

Questions

If you have any questions regarding this study, please contact:

Dr. Yasmine Hoydonckx (Principal Investigator) at the Comprehensive Integrated Pain Program, Interventional Pain Service: 416 603 2308

If you have any questions about your rights as a research participant or have concerns about this study, please call the Chair of the University Health Network Research Ethics Board (REB) or the Research Ethics office number at 416-581-7849. The REB is a group of people who oversee the ethical conduct of research studies. These people are not part of the study team. Everything that you discuss will be kept confidential.

Consent

I am willing for my participation to be communicated to my personal healthcare provider ☐ **YES** ☐ **NO**

This study has been explained to me and any questions I had have been answered. I know that I may leave the study at any time. I agree to take part in this study.

______________________	______________________	____________
Print Study Participant's Name	Signature	Date

(You will be given a signed copy of this consent form)

My signature means that I have explained the study to the participant named above. I have answered all questions.

______________________	______________________	____________
Print Name of Person Obtaining Consent	Signature	Date
